# Medical Items

**Published:** 1895-12

**Authors:** 


### *MEDICAL ITEMS.*

Dr. Nicholas Senn, of Chicago, Surgeon-General of Illinois, attended the Exposition, with the Governor's staff, Illinois day. While in the city Dr. Senn delivered an excellent clinical lecture at the Southern Medical College.

Dr. William H. V. Leyden, of Atlanta, died suddenly November 28th. A few years ago Dr. Leyden retired from the active duties of his profession. Formerly he enjoyed a large practice and was highly respected as an able and successful physician.

Says the *Journal de Medicine de Paris:* "We have serious reasons for believing that adhesion to exclusive bacillary pathology does not obtain better results than clinical medicine in the great majority of cases, and, moreover, compromises the constitutions of our patients."

Behring's law says that the blood and blood-serum of an individual which has been artificially rendered immune against a certain infectious disease, may be transferred into another individual with the effect to render the latter also immune, no matter how susceptible this individual is to the disease in question.—*Medical Record,* Sept. 14, 1895.

Pediatrics is the name of a new illustrated semi-monthly periodical devoted to the diseases of children. The first number is before us, dated January 1, 1896, containing some interesting papers and other matter relating to children's diseases. It is to be edited by Dr. Dillon Brown of New York, and Dr. George Carpenter of London. The first issue is very creditable.

THE United States Weather Bureau has begun the publication of a monthly entitled *Climate and Health.* It will be edited by Professor Willis L. Moore, Chief of the Weather Service, and Dr. W. F. R. Phillips. Two numbers have been issued containing interesting tables and charts relating to temperature, humidity, rainfall, mortality rates, and other important statistics.

---

A DEATH from the administration of ether occurred in this city November 30th. The patient was a healthy looking negro man, aged twenty-six, from whom a large tubercular inguinal gland was to be removed by Drs. A. R. Danforth and D. H. Howell. The ether was administered by Dr. Howell. A fresh can of Squibbs' ether was used, and about four ounces had been given when the patient's respiration ceased. The operation had not begun. Every effort was made to re-establish the respiration, but to no avail. The heart continued to beat for some minutes after the breathing stopped. We hope to be able to publish a full report of this case in our next issue.

---

THE first annual meeting of the Association of Southern Hospitals for the Insane convened in Atlanta, November 21st, lasting three days. The meeting was well attended by prominent representatives and superintendents of Southern hospitals for the insane, among whom were Dr. J. T. Searcy, of Tuscaloosa, Ala., President of the Association; Dr. J. W. Babcock, of Columbia, S. C., Secretary of the Association; Dr. Charles G. Hill, of Baltimore, Md., Vice-President; Dr. T. O. Powell of Milledgeville, Ga., Superintendent Georgia Insane Asylum; Dr. P. L. Murphy, of Morganton, N. C., Superintendent of the Western North Carolina Asylum; Dr. W. W. Godding, Superintendent of the Government Hospital for the Insane, of Washington, D. C.; Dr. R. J. Preston, Superintendent of the Western State Hospital, Marion, Va.; Dr.

George L. Kirby, Superintendent of the State Asylum, Raleigh, N. C.; Dr. Michael Campbell, Superintendent Eastern Hospital, Knoxville, Tenn.; Dr. F. H. Clarke, Superintendent Eastern Asylum, Lexington, Ky.; Dr. B. W. Stone, Superintendent Western Asylum, Hopkinsville, Ky.; Dr. J. J. Robertson, Superintendent State Asylum, Little Rock, Ark.; Dr. C. M. Rosser, Superintendent State Asylum, Terrell, Tex.; Dr. C. T. Simpson, Superintendent State Asylum, Austin, Tex.; Dr. J. M. Buchanan, Superintendent, and Dr. S. B. Watts, Secretary Board of Trustees State Asylum, Meridian, Miss., and others.

President Searcy, in his address, emphasized the rapid increase of insanity among civilized people, and suggested as etiological factors the enormous consumption of alcohol, tobacco, and the use of narcotics. He said that the "proper treatment of the insane most largely lies in the management they receive. Employment, diversion, and judicious discipline cure more patients than the medicines they take. The drug room, important as it is, does less good than the amusement hall, the work shops, and the farm. The brain is placed more at rest by judicious employment than by anodynes. Regular muscular exercise, in the very large majority of cases, brings more successfully than anything else, the other organs of the body to a normal condition, and the brain falls into line with the rest."

Among the papers presented and discussed was one on "Thyroid Feeding; its Action on the Insane," by Dr. C. G. Hill, Superintendent of Mount Hope Retreat, Baltimore; one by Dr. W. F. Drewry, of Virginia, on "Commitment of the Insane"; one by the Secretary, Dr. Babcock, on "The Feeble-minded"; one by Dr. T. O. Powell, of Georgia, on "The Colored Insane," etc.

The officers for the ensuing year are: Dr. Charles G. Hill, President; Dr. T. O. Powell, Vice-President, and Dr. J. W. Babcock, Secretary. Next meeting will be held in Asheville, N. C.

